# Feasibility and Outcomes of Laparoscopic Pancreatic Resections: Early Institutional Experience From a Resource-Limited Setting

**DOI:** 10.7759/cureus.97484

**Published:** 2025-11-22

**Authors:** Mariam Tahir Siddiqi, Sayyeda Zainab, Rimsha Farooq, Muhammad Hadis Saeed, Hunzla A Wajid, Zain Tayyab, Nighat Bakhtiar, Hafiza Sobia Ramzan, Syed Tatheer Abbas

**Affiliations:** 1 General Practice, Al-Aleem Medical College, Gulab Devi Teaching Hospital, Lahore, PAK; 2 General Medicine, District Headquarter Hospital, Narowal, PAK; 3 General Practice, Air University, Islamabad, PAK; 4 Surgery, Shaukat Khanum Memorial Cancer Hospital and Research Centre, Lahore, PAK; 5 Neurosurgery, Shaikh Zayed Medical Complex, Lahore, PAK; 6 Surgical Oncology, Shaukat Khanum Memorial Cancer Hospital and Research Centre, Lahore, PAK; 7 Gastroenterology, Cancer Care Hospital and Research Centre, Lahore, PAK; 8 Biochemistry, Institute of Biochemistry and Biotechnology, University of Lahore, Lahore, PAK

**Keywords:** better outcomes, laparoscopic surgery, pancreatic resection, patient-centred care, surgery

## Abstract

Background

Minimally invasive approaches have transformed many areas of abdominal surgery, but their application to pancreatic resections remains limited due to technical challenges and concerns regarding safety.

Objective

This study’s objective is to evaluate the feasibility, safety, and short-term outcomes of laparoscopic pancreatic resections during the initial institutional experience.

Methods

This retrospective observational study was conducted at Shaukat Khanum Memorial Cancer Hospital and Research Center from April 2022 to June 2025. It included 33 patients who underwent laparoscopic pancreatic resections. Data were collected on demographics, operative variables, postoperative outcomes, and histopathology. Outcomes assessed included operative time, blood loss, conversion to open surgery, complications, hospital stay, and margin status.

Results

The mean age of patients was 50.2 ± 14.8 years, with 30.3% (n = 10) being male. Distal pancreatectomy was the most common procedure, performed in 66.7% (n = 22) of patients, followed by enucleation in 21.2% (n = 7) and central pancreatectomy in 12.1% (n = 4). The mean operative time was 285 ± 45 minutes, with a mean blood loss of 210 ± 75 mL. Conversion to open surgery was required in 15.2% (n = 5) of cases. Overall, 33.3% (n = 11) of patients developed postoperative complications, with pancreatic fistula occurring in 12.1% (n = 4). The majority of complications were Clavien-Dindo grades I-II in 24.2% (n = 8) of patients. The mean hospital stay was 6.4 ± 2.1 days. Thirty-day mortality occurred in 3.0% (n = 1) of patients. Histopathology revealed pancreatic ductal adenocarcinoma in 30.3% (n = 10), neuroendocrine tumors in 24.2% (n = 8), and mucinous cystadenoma in 15.2% (n = 5). An R0 resection margin was achieved in 90.9% (n = 30) of malignant cases.

Conclusion

Laparoscopic pancreatic resections are feasible and safe in selected patients within a resource-limited setting. Operative outcomes, complication rates, and oncological adequacy in this early experience were comparable to international series.

## Introduction

Pancreatic surgery represents one of the most complex domains in abdominal surgery due to the gland’s deep retroperitoneal position, fragile parenchyma, and proximity to vital vascular structures such as the superior mesenteric vessels, portal vein, and splenic vessels [1]. During the past several decades, open surgery was regarded as the safest and most reliable technique when it comes to pancreatic resections, especially in the case of malignancies, due to the ability to clearly see, feel, and manipulate the major vessels [2]. However, the widespread development of less invasive procedures has progressively transformed the surgical scene, not just in the area of cholecystectomy and colorectal resection but even in the most daunting field of hepatopancreatobiliary surgery. In the 1990s, laparoscopic pancreatic resections were first performed, with Gagner and Pomp performing the laparoscopic pancreaticoduodenectomy in 1994 [3]. Soon after, laparoscopic distal pancreatectomy was reported and has since become the most commonly performed laparoscopic procedure on the pancreas. Firstly, these operations were met with a degree of skepticism because of doubts on technical feasibility, high pancreatic fistula rates, and the lack of understanding of the safety of the procedure in terms of oncology [4]. However, improvements in surgical technology and refinement of laparoscopic instruments and better energy devices over time have led to the ease of effecting safer and more effective resections. Emerging endoscopic staplers, vessel-sealing machines, and improved laparoscopic imaging have also led to the actualization of the minimally invasive pancreatic surgery [5].

Out of the various forms of laparoscopic resections of the pancreas, the distal pancreatectomy is deemed to be the most viable. This is due to the fact that the pancreatic body and tail are more reachable, and procedures can occasionally be carried out without necessarily involving a complicated reconstructive biliary or gastrointestinal work [6]. Small benign pancreatic tumors undergoing laparoscopic enucleation are also on the rise, especially in conditions such as insulinomas. More recently, selected very specialized centers have tried and achieved promising results with laparoscopic pancreaticoduodenectomy and even total pancreatectomy, but such surgeries are technically challenging and have not been widely adopted so far because of the steep learning curve [7]. Originally, laparoscopic pancreatic resections seem to have several benefits compared to open resection. Minimally invasive surgery has been associated with patients experiencing reduced intraoperative blood loss, less postoperative pain, shorter hospitalization, and quicker recovery to normal activities. Moreover, cosmetic advantage is another factor, particularly among younger patients [8]. Notably, research has established that laparoscopic distal pancreatectomy offers similar oncological results compared to open resection in terms of resection margins, the number of lymph nodes, and long-term survival in pancreatic cancer. Although these may be the possible advantages, there are major challenges [9]. Laparoscopic pancreatic surgery is linked to a steep learning curve, with research indicating that a surgeon might need 40-60 cases to master the laparoscopic distal pancreatectomy, in addition to more to master pancreaticoduodenectomy [10]. The duration of operation is usually prolonged, especially during the initial adoption period, but it tends to reduce over time [11].

## Materials and methods

This retrospective observational study was conducted at Shaukat Khanum Memorial Cancer Hospital and Research Center from April 2022 to June 2025. A total of 33 patients who met the eligibility criteria were included. This study included patients aged 18 years and above who underwent laparoscopic pancreatic resections, including distal pancreatectomy, central pancreatectomy, or enucleation. Only those with histologically confirmed benign, borderline, or malignant pancreatic lesions deemed suitable for a laparoscopic approach were enrolled. Patients were excluded if they had undergone open pancreatic resections, required emergency pancreatic surgery, or had locally advanced pancreatic disease involving major vascular structures.

Data collection

Patient records, operative notes, and hospital databases were reviewed to extract demographic data (age, gender, BMI), presenting symptoms, radiological findings, type of surgery performed, operative time, estimated blood loss, conversion to open surgery, and intraoperative complications. Postoperative information such as length of stay in hospital, postoperative morbidity and mortality, and 30-day readmission rates were also obtained. The complications were categorized using the Clavien-Dindo grading system, and the incidence of clinically relevant postoperative pancreatic fistula was recorded using the International Study Group of Pancreatic Surgery (ISGPS) definition. Histopathological information was taken, including the size of the tumor, diagnosis, and the status of its resection margin. Surgeons who had undergone advanced training in minimally invasive surgery carried out all the procedures through the use of standard laparoscopic procedures. Depending on the nature of the procedure, four to five laparoscopic ports were introduced. Endoscopic staplers or energy devices were used to perform pancreatic transection. The splenic preservation was tried where possible during distal pancreatectomy. This was done by use of a Pfannenstiel or mini-laparotomy incision to retrieve the specimens in an endoscopic retrieval bag. The main outcomes evaluated were the time of the operation, perioperative blood loss, conversion to open operation, and postoperative complications.

Data analysis

Data were entered and analyzed using the Statistical Package for Social Sciences (SPSS) version 26.0 (IBM Corp., Armonk, NY). Quantitative variables such as age, operative time, blood loss, and length of hospital stay were expressed as mean ± standard deviation (SD). Categorical variables such as gender, type of procedure, conversion rate, and postoperative complications were presented as frequencies and percentages.

## Results

Data were collected from 33 patients; the mean age of the patients was 50.2 ± 14.8 years, and 10 (30.3%) were male. The average BMI was 24.7 ± 3.5 kg/m². Abdominal pain was the most frequent presenting symptom, seen in 21 patients (63.6%), while six patients (18.2%) were diagnosed incidentally. Jaundice was reported in four patients (12.1%), and other symptoms, including weight loss, were noted in two patients (6.1%). Distal pancreatectomy was the most common surgical procedure, performed in 22 patients (66.7%), followed by enucleation in seven patients (21.2%) and central pancreatectomy in four patients (12.1%). The mean operative time was 285 ± 45 minutes, with an estimated blood loss of 210 ± 75 mL. Conversion to open surgery was required in five patients (15.2%) (Table 1).

**Table 1:** Baseline Demographic, Clinical, and Operative Characteristics (n = 33) Demographic and clinical characteristics of patients, including age, gender, BMI, presenting symptoms, types of procedures, and operative details.

Characteristic	Value
Age (years), mean ± SD	50.2 ± 14.8
Gender (male), n (%)	10 (30.3%)
BMI (kg/m²), mean ± SD	24.7 ± 3.5
Presenting symptoms	
- Abdominal pain, n (%)	21 (63.6%)
- Incidental finding, n (%)	6 (18.2%)
- Jaundice, n (%)	4 (12.1%)
- Others, n (%)	2 (6.1%)
Type of procedure	
- Distal pancreatectomy, n (%)	22 (66.7%)
- Enucleation, n (%)	7 (21.2%)
- Central pancreatectomy, n (%)	4 (12.1%)
Operative time (minutes), mean ± SD	285 ± 45
Blood loss (mL), mean ± SD	210 ± 75
Conversion to open, n (%)	5 (15.2%)

The mean duration of hospital stay was 6.4 ± 2.1 days. Overall complications were observed in 11 patients (33.3%). Pancreatic fistula was the most common complication, occurring in four patients (12.1%). Delayed gastric emptying and intra-abdominal collections were each observed in two patients (6.1%), while wound infection was noted in one patient (3.0%). Most complications were minor (Clavien-Dindo grades I-II) and occurred in eight patients (24.2%). Severe complications (grades III-IV) developed in three patients (9.1%). There was one case of 30-day mortality (3.0%) (Table 2).

**Table 2:** Postoperative Complications, Length of Hospital Stay, and 30-Day Mortality (n = 33) Postoperative complications, hospital stay, and 30-day mortality, with complications categorized by Clavien-Dindo grade.

Outcome	Value
Hospital stay (days), mean ± SD	6.4 ± 2.1
Overall complications, n (%)	11 (33.3%)
Pancreatic fistula, n (%)	4 (12.1%)
Delayed gastric emptying, n (%)	2 (6.1%)
Intra-abdominal collection, n (%)	2 (6.1%)
Wound infection, n (%)	1 (3.0%)
Clavien-Dindo grades I-II, n (%)	8 (24.2%)
Clavien-Dindo grades III-IV, n (%)	3 (9.1%)
30-day mortality, n (%)	1 (3.0%)

Histological evaluation revealed that pancreatic ductal adenocarcinoma was the most frequent diagnosis, observed in 10 patients (30.3%). Neuroendocrine tumors were found in eight patients (24.2%), while mucinous cystadenomas were identified in five patients (15.2%) and serous cystadenomas in three patients (9.1%). Solid pseudopapillary neoplasms accounted for four cases (12.1%), and other benign non-neoplastic lesions were found in three cases (9.1%). Importantly, margin-negative resection (R0) was achieved in 30 patients (90.9%) (Table 3).

**Table 3:** Histopathological Diagnosis and Margin Status of Pancreatic Lesions (n = 33) Histopathological diagnoses of pancreatic lesions and the R0 resection margin status for malignant cases.

Pathology	n (%)
Serous cystadenoma	3 (9.1%)
Mucinous cystadenoma	5 (15.2%)
Neuroendocrine tumor	8 (24.2%)
Pancreatic ductal adenocarcinoma	10 (30.3%)
Solid pseudopapillary neoplasm	4 (12.1%)
Others/benign non-neoplastic	3 (9.1%)
R0 margin achieved	30 (90.9%)

Complication severity analysis showed that pancreatic fistula occurred in four patients (12.1%), distributed as grade I in one patient, grade II in two patients, and grade III in one patient. Delayed gastric emptying occurred in two patients (6.1%), with one grade II and one grade III case. Intra-abdominal collections were observed in two patients (6.1%), including one grade II and one grade III complication. Wound infection was recorded in one patient (3.0%) as a grade I event. Other minor complications were noted in two patients (6.1%). In total, three patients (9.1%) had grade I, five (15.2%) had grade II, and three (9.1%) had grade III complications (Table 4).

**Table 4:** Severity of Postoperative Complications According to Clavien-Dindo Grading (n = 33) Postoperative complications by severity (Clavien-Dindo grades I-IV), with statistical analysis showing no significant differences in complication severity (p = 0.41). The association between the type and severity of postoperative complications was evaluated using the chi-square test. A p-value < 0.05 was considered the threshold for statistical significance in all analyses.

Complication Type	Grade I, n (%)	Grade II, n (%)	Grade III, n (%)	Grade IV, n (%)	Total, n (%)
Pancreatic fistula	1 (3.0%)	2 (6.1%)	1 (3.0%)	0	4 (12.1%)
Delayed gastric emptying	0	1 (3.0%)	1 (3.0%)	0	2 (6.1%)
Intra-abdominal collection	0	1 (3.0%)	1 (3.0%)	0	2 (6.1%)
Wound infection	1 (3.0%)	0	0	0	1 (3.0%)
Others	1 (3.0%)	1 (3.0%)	0	0	2 (6.1%)
Total	3 (9.1%)	5 (15.2%)	3 (9.1%)	0	11 (33.3%)

Patients with malignant lesions were significantly older than those with benign lesions (55.2 ± 11.7 vs. 44.8 ± 12.6 years, p = 0.03). Operative time was also longer in malignant cases (298 ± 42 minutes vs. 270 ± 38 minutes, p = 0.04), and mean blood loss was greater (235 ± 80 mL vs. 185 ± 70 mL, p = 0.05). Conversion to open surgery occurred more frequently in malignant lesions (22.2% vs. 6.7%), although this was not statistically significant. Complication rates were higher in the malignant group (38.9% vs. 26.7%), but the difference was not significant (p = 0.41). Pancreatic fistula occurred in 2 patients in each group, showing no major difference. Hospital stay was slightly longer for malignant cases (6.8 ± 2.2 vs. 5.9 ± 1.8 days). One 30-day mortality was recorded in the malignant group (5.6%), while no deaths occurred in the benign group (Table 5).

**Table 5:** Comparison of Surgical Outcomes Between Benign and Malignant Pancreatic Lesions Outcomes between benign and malignant lesions, showing significant differences in age, operative time, and blood loss (p < 0.05). Statistical analysis used Student’s t-test and chi-squared test.

Variable	Benign (n = 15)	Malignant (n = 18)	p-value
Age (years), mean ± SD	44.8 ± 12.6	55.2 ± 11.7	0.03
Operative time (minutes), mean ± SD	270 ± 38	298 ± 42	0.04
Blood loss (mL), mean ± SD	185 ± 70	235 ± 80	0.05
Conversion to open, n (%)	1 (6.7%)	4 (22.2%)	0.18
Postoperative complications, n (%)	4 (26.7%)	7 (38.9%)	0.41
Pancreatic fistula, n (%)	2 (13.3%)	2 (11.1%)	0.82
Hospital stay (days), mean ± SD	5.9 ± 1.8	6.8 ± 2.2	0.12
30-day mortality, n (%)	0	1 (5.6%)	0.29

## Discussion

This study presents the initial institutional experience of laparoscopic pancreatic resections in 33 patients. We have shown that it is possible to perform minimal invasive pancreatic surgery in a resource-restricted environment and with acceptable operative times, complications, and oncology outcomes. These outcomes reflect the trends found in other nations and are congruent with the initial outcomes in other laparoscopic distal pancreatectomy units in Pakistan, such as Shaukat Khanum, which has already reported a smaller series of laparoscopic distal pancreatectomies. The most common procedure in our series was distal pancreatectomy (66.7%), which is consistent with practice in the rest of the world, as distal resection is the starting point of laparoscopic pancreatic surgery because it is technically relatively simple. Our operative time of 285 was longer than the conventional open resections but similar to other early laparoscopic experiences, with operation time frequently longer than 250-300 minutes during the learning curve. Equally, we had an average blood loss (210 mL) within international reported limits and less than the conventional open pancreatic resections [12]. The rate of conversion to open surgery was 15.2%, which showed the anticipated difficulties in the early stage of adoption. The laparoscopic distal pancreatectomy experience of Shaukat Khanum gave a similar conversion rate of 20% indicating that our findings are comparable to those of other early Pakistani series. Our total complication rate of 33.3 is in the accepted international range (25-40) of pancreatic surgery [13].

The pancreatic fistula rate of 12.1% in the present cohort corresponds to the already reported rates of 10-20% fistula rates, depending on the definition of fistula and the surgical procedure. Significantly, most of the complications were low grade (Clavien-Dindo I-II) and could be treated by conservative methods. The steep learning curve of laparoscopic pancreatic resections is demonstrated by the relatively high conversion rate and length of operation. Evidence published indicates that, on average, a surgeon needs about 40-60 cases before becoming skilled in laparoscopic distal pancreatectomy, and even more before becoming skilled in more complex procedures, such as laparoscopic pancreaticoduodenectomy. The outcomes of our results are also indicative of the initial phase of institutional experience and will be expected to get better as the number of cases and systematic training increases [14]. Our results are in the expected ranges of early results compared to the international series. This was supported by a large multi-institutional review of Europe and North America with reported conversion rates of 10-25, mean operative time of 270-320 minutes, and complication rates of 30-40, all similar to our findings. Likewise, local experience at Shaukat Khanum has shown that laparoscopic pancreatic resections can be implemented in Pakistan with reasonable morbidity and mortality, but low volumes. Our 33-patient study is one of the larger pilot experiences in the area and contributes to the small South Asian data on the topic [15,16].

Nevertheless, there are a number of limitations that need to be considered. To begin with, the retrospective design has some intrinsic risks of a lack of data and reporting bias. Second, the sample size is quite small, which restricts the statistical power and generalizability. Third, there was a short duration of follow-up, which did not allow evaluation of long-term oncological events, including recurrence and survival. Lastly, we only had a range of cases that would be suitable for laparoscopic resection, and findings might not be generalized to less laparoscopic and more complex resections like pancreaticoduodenectomy.

## Conclusions

It is concluded that laparoscopic pancreatic resections are feasible and safe in carefully selected patients, even during the initial phase of institutional experience. The procedures were associated with acceptable operative times, manageable blood loss, and complication rates comparable to those reported in international literature. Oncological adequacy was achieved in the majority of malignant cases, with a high rate of margin-negative resections.
